# Comparison of logistic regression and machine learning methods for predicting early neurological deterioration after thrombolysis in patients with mild stroke

**DOI:** 10.3389/fneur.2026.1703890

**Published:** 2026-03-04

**Authors:** Chen Lou, Meiyun Zhang, Jingjing Li, Dongjuan Xu

**Affiliations:** Department of Neurology, Dongyang People’s Hospital, Affiliated to Wenzhou Medical University, Dongyang, Zhejiang, China

**Keywords:** early neurological deterioration, intravenous thrombolysis, machine learning, mild ischemic stroke, prediction model

## Abstract

**Background:**

We aimed to explore the risk factors for early neurological deterioration after thrombolysis in patients with mild stroke. Machine learning model and logistic regression model were established. We compared them to facilitate early identification of patients with mild stroke who still experience early neurological deterioration after thrombolysis. It can alert the physician and clinical remedial measures can be prepared in advance.

**Methods:**

We conducted a study on patients with mild stroke who underwent thrombolysis from April 1, 2017 to April 1, 2024 at emergency department. Four common machine learning methods-Extreme Gradient Boosting (XGBoost), K-Nearest Neighbors (KNN), Random Forest (RF), and Support Vector Machine (SVM)-were used to create predictive models based on the information of eligible participants. The unbalanced data was preprocessed using four different methods. Each machine learning model was paired with four preprocessing schemes, resulting in 16 workflows. Then, we selected the optimal machine learning model from them. Additionally, five methods were used to establish logistic regression models. The optimal logistic regression model was then selected from them.

**Results:**

A total of 625 patients with mild stroke were included in the study, among whom 80 experienced early neurological deterioration after thrombolysis. Through 10-fold stratified cross-validation and simulated annealing algorithm, the optimal model among the four machine learning methods was selected as the SVM model that balanced the data through upsampling in 16 workflows. The area under the curve (AUC) of the SVM model was 0.889 (95% CI: 0.853, 0.926) in the training set and 0.859 in the test set processed by upsampling. Among the five methods used to establish logistic regression models, model m4 was the optimal one, with an AUC of 0.848 in the test set.

**Conclusion:**

We explored the risk factors influencing the occurrence of early neurologic deterioration after thrombolysis in patients with mild stroke. We also found that logistic regression model and machine learning model demonstrated comparable performance in this single-center retrospective dataset.

## Introduction

In clinical practice, more than half of patients with ischemic stroke exhibit a clinical syndrome characterized by mild neurological deficits. In the United States, 4 out of every 10 patients receiving intravenous thrombolytic therapy have mild deficits (1). Recombinant tissue plasminogen activator (rt-PA) intravenous thrombolysis (IVT) has been proven to be an effective treatment for acute ischemic stroke, but its benefits and risks for mild strokes remain unclear. Some studies have reported that 9% of patients with mild strokes experienced early neurological deterioration despite receiving intravenous thrombolysis (2).

Age, diabetes, hypertension, intracranial or extracranial vascular occlusive lesions, and NIHSS scores have been shown to be associated with poor outcomes in patients with mild stroke (3–5). However, most of these studies were based on traditional logistic regression models and focused on factors associated with disability at 90 days in patients with mild stroke who did not receive intravenous thrombolysis. Exacerbation in mild stroke patients after thrombolysis is a current therapeutic challenge. It is important to early identify patients with mild stroke who experience neurological deterioration after thrombolysis. It can reduce the rates of disability and mortality.

Machine learning (ML) is a form of artificial intelligence that enables software applications to predict outcomes more accurately without explicit programming. Due to its ability to analyze underlying mechanisms of varying complexity, it is currently widely used in various clinical fields to predict events of interest. However, there is still debate as to whether complex machine learning algorithms can surpass traditional models in specific fields. We compared the performance of traditional logistic regression and machine learning methods in predicting early neurological deterioration in patients with mild stroke after thrombolysis. These models can alert the physician. Clinical remedial measures can be prepared in advance.

## Materials and methods

### Study population

We included patients with cerebral infarction who received intravenous thrombolysis of rt-PA at the emergency department of Dongyang People’s Hospital from April 1, 2017 to April 1, 2024. Patients with mild stroke (NIHSS score of 0–5 points) at admission were selected among them. Exclusion criteria consisted of: (1) patients with contraindications for intravenous thrombolysis according to the IVT standard guidelines (6); (2) patients who received endovascular therapy combined with intravenous thrombolysis; (3) patients who experienced bleeding after thrombolysis; (4) patients with missing data.

### Data collection

**Figure 1:**
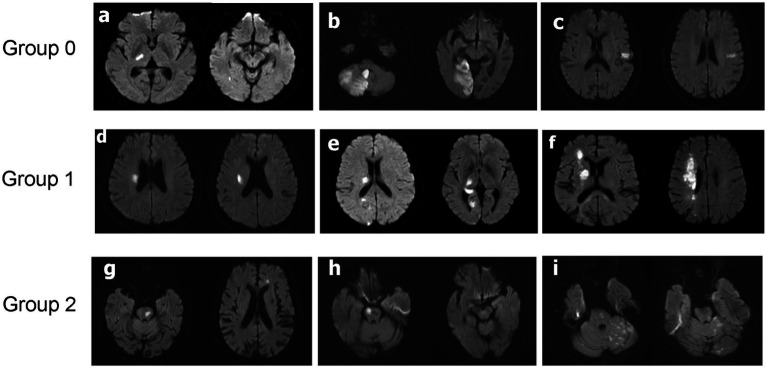
Three different groups of DWI cerebral infarction patterns. Group 0: thalamic infarcts **(a)**, cerebellar infarcts **(b)**, temporal lobe infarcts **(c)**. Group 1: lateral paraventricular infarcts **(d)**, lateral paraventricular combined with additional infarcts elsewhere **(e)**, basal ganglia infarcts combined with other sites additional **(f)**. Group 2: pontine infarcts combined with other sites additional infarction **(g,i)**, pontine infarction **(h)**.

This retrospective study collected baseline characteristics of patients, including demographic data (gender, age, alcohol consumption, smoking), medical history (diabetes, hypertension, previous atrial fibrillation, previous coronary heart disease, previous stroke), medications prior to stroke (antiplatelet agents, plaque-stabilizing agents), NIHSS score at admission (0–2 points or 3–5 points), systolic blood pressure prior to thrombolysis, diastolic blood pressure prior to thrombolysis, time from onset to thrombolysis (OTT), imaging studies (to determine the location of cerebral infarction and TOAST type via head MRI and cranial and cervical CTA), and laboratory tests (whether high-sensitivity C-reactive protein (hs-CRP) > 5 mg/L, red blood cell count, hemoglobin, white blood cell count, neutrophil count, lymphocyte count, monocyte count, neutrophil-to-lymphocyte ratio (NLR), platelet count, serum potassium, serum sodium, serum chloride, serum calcium, blood glucose, glycated hemoglobin, blood urea nitrogen, creatinine, prothrombin time (PT), activated partial thromboplastin time (APTT), international normalized ratio (INR), fibrinogen (FIB), total cholesterol (TC), high-density lipoprotein (HDL), low-density lipoprotein (LDL), triglycerides (TG), TG/HDL, D-dimer after thrombolysis). Head MRI, partial cranial and cervical CTA results were obtained within 24–48 h after thrombolysis. Because the following parameters—hs-CRP > 5 mg/L, glycated hemoglobin, HDL, LDL, TC, TG, TG/HDL, and D-dimer after thrombolysis—were not tested in the emergency department, these parameters were obtained within 24 h after admission to the ward following emergency department admission. All other laboratory parameters were available upon admission to the emergency department. In most cases, rt-PA was administered according to the standard protocol: a dose of 0.9 mg/kg, with a total dose not exceeding 90 mg, administered over 60 min, with an initial dose of 10% of the total dose administered in the first minute. However, a small proportion of patients received rt-PA according to different guidelines: the dose is 0.6 mg/kg, with a maximum total dose of 90 mg over 60 min, but the initial dose in the first minute was 15% of the total dose (7). Stroke subtypes were determined by investigators using an expanded version of the TOAST classification (ORG10172 trial in acute stroke treatment), with cranial and cervical CTA used to assess arterial stenosis or occlusion. Based on the etiology of cerebral infarction, cases caused by cerebral artery stenosis or occlusion were defined as TOAST-B group, while cases with small-artery occlusion lacunar, cardiac etiology, other etiologies, or unknown etiology were defined as TOAST-A group. Based on head MRI results, lesions involving the lateral ventricle region and basal ganglia were classified as Group 1, lesions involving the brainstem were classified as Group 2, and lesions not involving the lateral ventricle region, basal ganglia, or brainstem were classified as Group 0 (Figure 1).

### Definitions of outcomes

This study investigated early neurological deterioration after intravenous thrombolysis in mild stroke, with neurological function assessed using the National Institutes of Health Stroke Scale (NIHSS) (8). Early neurological deterioration (END) was defined as an increase of 2 points or more in the NIHSS score within 24 h of thrombolysis (exclude cerebral hemorrhage).

### Construction of machine learning models

625 patients were randomly divided into training and testing groups at a ratio of 8:2. Stratified sampling was performed to ensure that the proportions of each category were consistent in the training and testing sets. Due to the limited number of patients with early neurological deterioration after thrombolysis, we addressed the imbalanced data by creating four different data preprocessing recipes for the training set variables to enhance predictive performance: (1) No resampling (Base); (2) Upsampling of the minority class to balance category proportions; (3) Downsampling of the majority class to reduce its sample size. (4) Using the Synthetic Minority Oversampling Technique (SMOTE) to synthesize minority class samples. This study examined four powerful machine learning models, including Extreme Gradient Boosting (XGBoost), k-Nearest Neighbors (KNN), Random Forest (RF), and Support Vector Machines (SVM). Each machine learning model was paired with four preprocessing schemes, resulting in 16 workflows. These workflows were used to integrate preprocessing steps and model specifications for systematic model training and evaluation. Each machine learning model incorporated the same set of variables. All variables were included without prior feature selection. These variables included demographic data (gender, age, alcohol consumption, smoking), medical history (diabetes, hypertension, atrial fibrillation, coronary heart disease, stroke), medications prior to stroke (antiplatelet agents, plaque-stabilizing agents), NIHSS score, systolic blood pressure, diastolic blood pressure, OTT, imaging studies (head MRI and cranial and cervical CTA), and laboratory tests (CRP, red blood cell count, hemoglobin, white blood cell count, neutrophil count, lymphocyte count, monocyte count, NLR, platelet count, serum potassium, serum sodium, serum chloride, serum calcium, blood glucose, glycated hemoglobin, blood urea nitrogen, creatinine, PT, APTT, INR, FIB, TC, HDL, LDL, TG, triglycerides/high-density lipoprotein, D-dimer). All models were evaluated using 10-fold stratified cross-validation to ensure the reproducibility of results. In 10-fold stratified cross-validation, we ensured that the proportions of the early neurological deterioration group and the non-deterioration group after thrombolysis remained consistent across each fold, thereby enhancing the robustness of model evaluation. The 16 workflows were optimized using a simulated annealing algorithm. In the context of highly imbalanced datasets, we selected AUC to compare model performance because it comprehensively evaluated the overall performance of a model across different thresholds, unaffected by class distribution imbalances. The optimal workflow with the highest area under the curve (AUC) was selected through 10-fold stratified cross-validation and simulated annealing algorithm. The AUC, specificity, sensitivity, accuracy, positive predictive value (PPV), negative predictive value (NPV), precision, recall, and F1 score were calculated in the training and testing sets. To gain an intuitive understanding of the essence of machine learning models with “black box” characteristics, this study introduced the SHAP framework to explain the optimal machine learning model. Its interpretability performance has been validated in many models.

### Construction of logistic regression models

Using an 8:2 ratio, patients were randomly divided into training group and test group. Stratified sampling was also performed to ensure that the proportions of each category were consistent in the training set and test set. In the training set, model m0 was constructed using predictor variables with statistically significant intergroup differences (*p* < 0.05). Model m1 was constructed using all predictive variables. Model m2 was constructed after variable selection using the backward method. Model m3 was constructed after variable selection using the stepwise method. Model m4 was reconstructed using the predictive variables that were significantly associated through the aforementioned methods (*p* < 0.05). The best model was selected by comparing the *p*-values of the ANOVA tests and the number of variables among the five models. A nomogram was established to predict early neurological deterioration after IVT in mild stroke using the best logistic model.

### Statistical analysis

Continuous data and categorical data were expressed as median (interquartile range) and proportion, respectively. Intergroup comparisons between the early neurological deterioration group and the non-deterioration group after thrombolysis were performed using unpaired t-tests or Wilcoxon rank-sum tests, Pearson chi-square tests, or Fisher’s exact tests as appropriate. R software (version 4.3.3; R Foundation for Statistical Computing, Vienna, Austria) was used. *p* < 0.05 was considered statistically significant.

## Results

### Baseline characteristics

**Figure 2:**
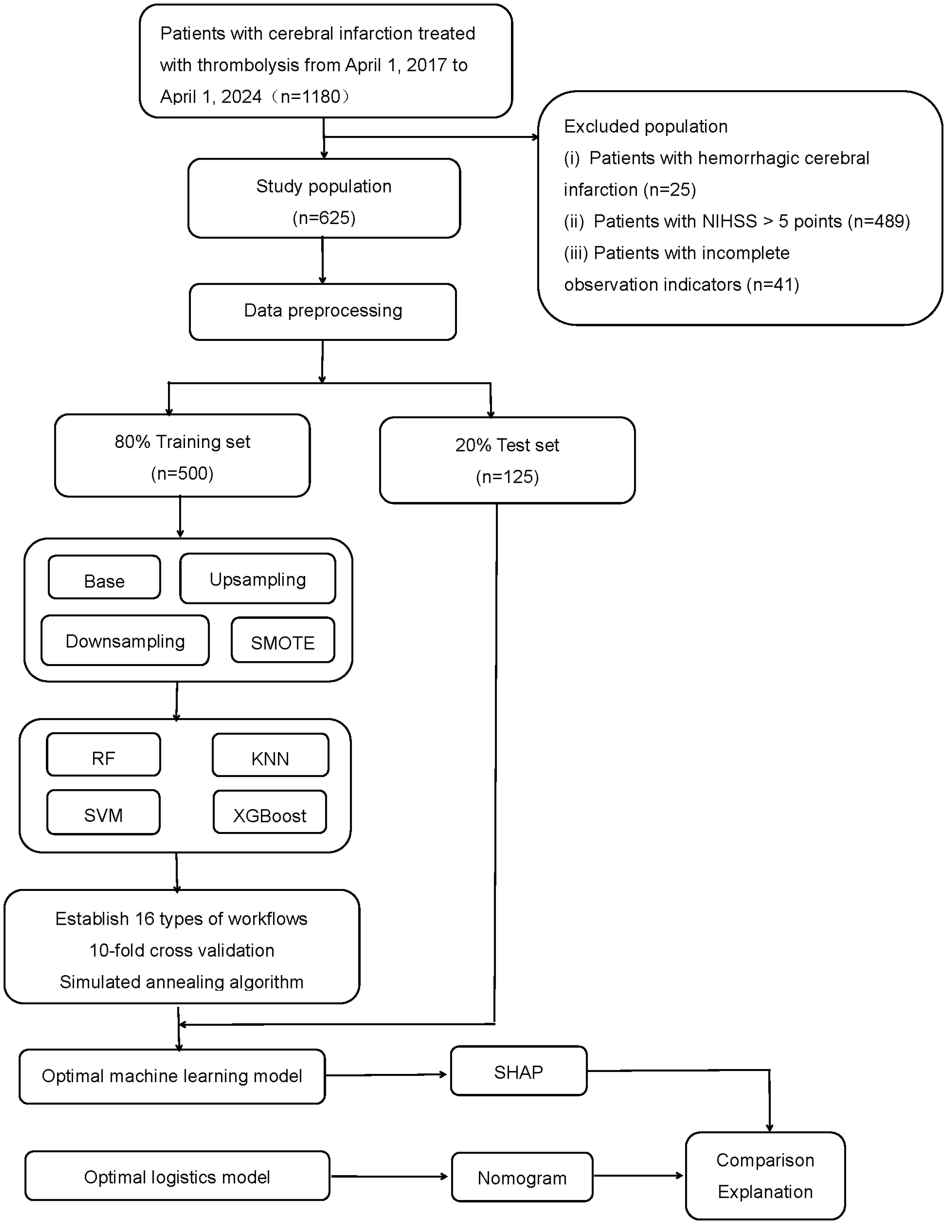
The flow chart of patients selection and the development, evaluation, and explanation of models. Base: without any resampling; Upsampling: upsampling of the minority class to balance category proportions; Downsampling: downsampling of the majority class to reduce its sample size; SMOTE: synthesizing a few class samples. XGBoost: Extreme Gradient Boosting; KNN: *K*-nearest neighbors; RF: random forest; SVM: support vector machine.

We included a total of 1,180 patients with thrombolysis for cerebral infarction. 625 mild stroke patients were included. Among them, 500 individuals were assigned to the training set, and the remaining 125 individuals formed the test set in an 8:2 ratio (Figure 2). Among the 625 patients included in the study, 80 patients with mild cerebral infarction who underwent thrombolysis experienced early neurological deterioration, while 545 patients did not experience early neurological deterioration. The baseline characteristics of the early neurological deterioration group and the non-early neurological deterioration group were shown in Table 1.

**Table 1:** Demographic and clinical characteristics of study participants.

Variables	Non-END *N* = 545	END *N* = 80	*p*-value
Gender, *n* (%)			0.1
Female	181 (33%)	34 (43%)	
Male	364 (67%)	46 (58%)	
Age(years), median (Q1, Q3)	68 (59, 75)	70 (57, 77)	>0.9
Smoking (any cigarettes/week), *n* (%)	238 (44%)	37 (46%)	0.7
Alcohol use (≥ one unit/day), *n* (%)	239 (44%)	40 (50%)	0.3
Hypertension, *n* (%)	385 (71%)	54 (68%)	0.6
Diabetes mellitus, *n* (%)	111 (20%)	18 (23%)	0.7
Atrial fibrillation, *n* (%)	64 (12%)	5 (6.3%)	0.14
Coronary heart disease, *n* (%)	68 (12%)	2 (2.5%)	0.008
History of stroke, *n* (%)	83 (15%)	10 (13%)	0.5
Antiplatelet, *n* (%)	69 (13%)	3 (3.8%)	0.02
Statins, *n* (%)	67 (12%)	2 (2.5%)	0.009
NIHSS, *n* (%)			<0.001
NIHSS 0–2	230 (42%)	7 (8.8%)	
NIHSS 3–5	315 (58%)	73 (91%)	
Onset to rt-PA time, median (Q1, Q3)	150 (100, 200)	140 (100, 180)	0.11
TOAST, *n* (%)			<0.001
TOAST-A	455 (83%)	50 (63%)	
TOAST-B	90 (17%)	30 (38%)	
SBP(mmHg), median (Q1, Q3)	152 (139, 166)	156 (140, 171)	0.093
DBP(mmHg), median (Q1, Q3)	83 (73, 93)	84 (75, 92)	0.8
DWI lesion pattern, *n*(%)			<0.001
Group 0	393 (72%)	14 (18%)	
Group 1	111 (20%)	48 (60%)	
Group 2	41 (7.5%)	18 (23%)	
Laboratory data, median (Q1, Q3)			
RBC (*10 ^12^/L)	4.60 (4.29, 4.95)	4.67 (4.35, 5.04)	0.4
Hemoglobin (g/L)	144 (133, 154)	142 (131, 154)	0.5
WBC(*10 ^9^/L)	7.05 (5.88, 8.46)	7.38 (6.28, 9.21)	0.039
Neutrophil (*10 ^9^/L)	4.27 (3.44, 5.52)	4.61 (3.73, 6.64)	0.028
Lymphocyte (*10 ^9^/L)	1.88 (1.44, 2.59)	1.86 (1.41, 2.40)	0.5
Monocyte (*10 ^9^/L)	0.46 (0.37, 0.58)	0.41 (0.34, 0.55)	0.1
NLR	2.15 (1.54, 3.30)	2.38 (1.62, 4.00)	0.094
Platelet (*10 ^9^/L)	208 (170, 243)	208 (171, 265)	0.3
PT(s)	13.00 (12.50, 13.50)	13.00 (12.60, 13.40)	>0.9
APTT(s)	34.0 (31.9, 36.6)	34.1 (30.7, 36.9)	0.7
INR	0.99 (0.95, 1.04)	0.99 (0.96, 1.04)	0.7
Fibrinogen (g/L)	3.18 (2.73, 3.69)	3.34 (2.95, 3.85)	0.029
Potassium (mmol/L)	3.82 (3.59, 4.05)	3.77 (3.53, 4.08)	0.5
Sodium (mmol/L)	139.10 (137.60, 141.00)	139.00 (137.50, 141.00)	0.7
Chlorinum (mmol/L)	103.3 (101.0, 105.7)	103.5 (100.9, 105.1)	>0.9
Calcium (mmol/L)	2.29 (2.23, 2.36)	2.28 (2.24, 2.34)	0.4
Blood glucose level (mmol/L)	6.83 (6.02, 8.51)	7.58 (6.29, 9.49)	0.024
BUN (mmol/L)	5.60 (4.80, 6.90)	5.55 (4.62, 6.65)	0.3
Serum creatinine (μmol/L)	72 (61, 85)	69 (57, 84)	0.14
D-dimer after thrombolysis (mg/L)	0.96 (0.48, 2.12)	1.06 (0.45, 2.24)	>0.9
HbAlc(%)	5.80 (5.40, 6.30)	5.80 (5.50, 6.40)	0.6
TC (mg/dL)	4.29 (3.63, 4.90)	4.53 (3.90, 5.18)	0.022
TG (mg/dL)	1.26 (0.92, 1.94)	1.30 (0.90, 1.87)	>0.9
LDL (mg/dL)	2.52 (1.95, 3.11)	2.92 (2.58, 3.44)	<0.001
HDL (mg/dL)	1.05 (0.89, 1.25)	1.04 (0.86, 1.25)	0.8
TG/HDL	1.23 (0.78, 1.93)	1.21 (0.80, 1.73)	>0.9
hs-CRP, *n*(%)			<0.001
Lower than 5 mg/L	427 (78%)	48 (60%)	
Higher than 5 mg/L	118 (22%)	32 (40%)	

Data are presented as *N* (%) or median (interquartile range). NIHSS, National Institutes of Health Stroke Scale; TOAST-A, small-artery occlusion lacunar or cardio embolism or acute stroke of other determined etiology or stroke of other undetermined etiology; TOAST-B, large-artery atherosclerosis; SBP, systolic blood pressure; DBP, diastolic blood pressure; RBC, red blood cell; WBC, white blood cell; NLR, neutrophil-lymphocyte ratio; PT, prothrombin time; APTT, active partial thromboplastin; INR, International normalized ratio; BUN, blood urea nitrogen; TC, total cholesterol; TG, triglyceride; HDL, high-density lipoprotein; LDL, low-density lipoprotein; TG/HDL, triglyceride to high-density lipoprotein ratio; DWI lesion pattern Group 0, no infarction in brain stem, paraventricular and basal ganglia; DWI lesion pattern Group 1, paraventricular infarction or basal ganglia infarction with additional infarcts outside these areas; DWI lesion pattern Group 2, brainstem infarction with additional infarcts outside the brainstem territory; hs-CRP, high-sensitivity C-reactive protein.

### Comparison of various machine learning models

**Figure 3:**
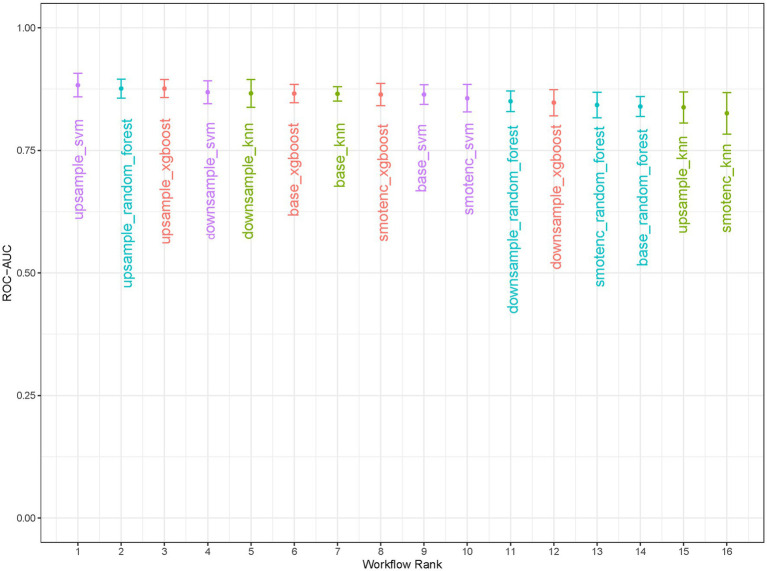
Comparison of 16 workflow types. XGBoost, RF, SVM, KNN were processed by four different ways of handling data.

**Figure 4:**
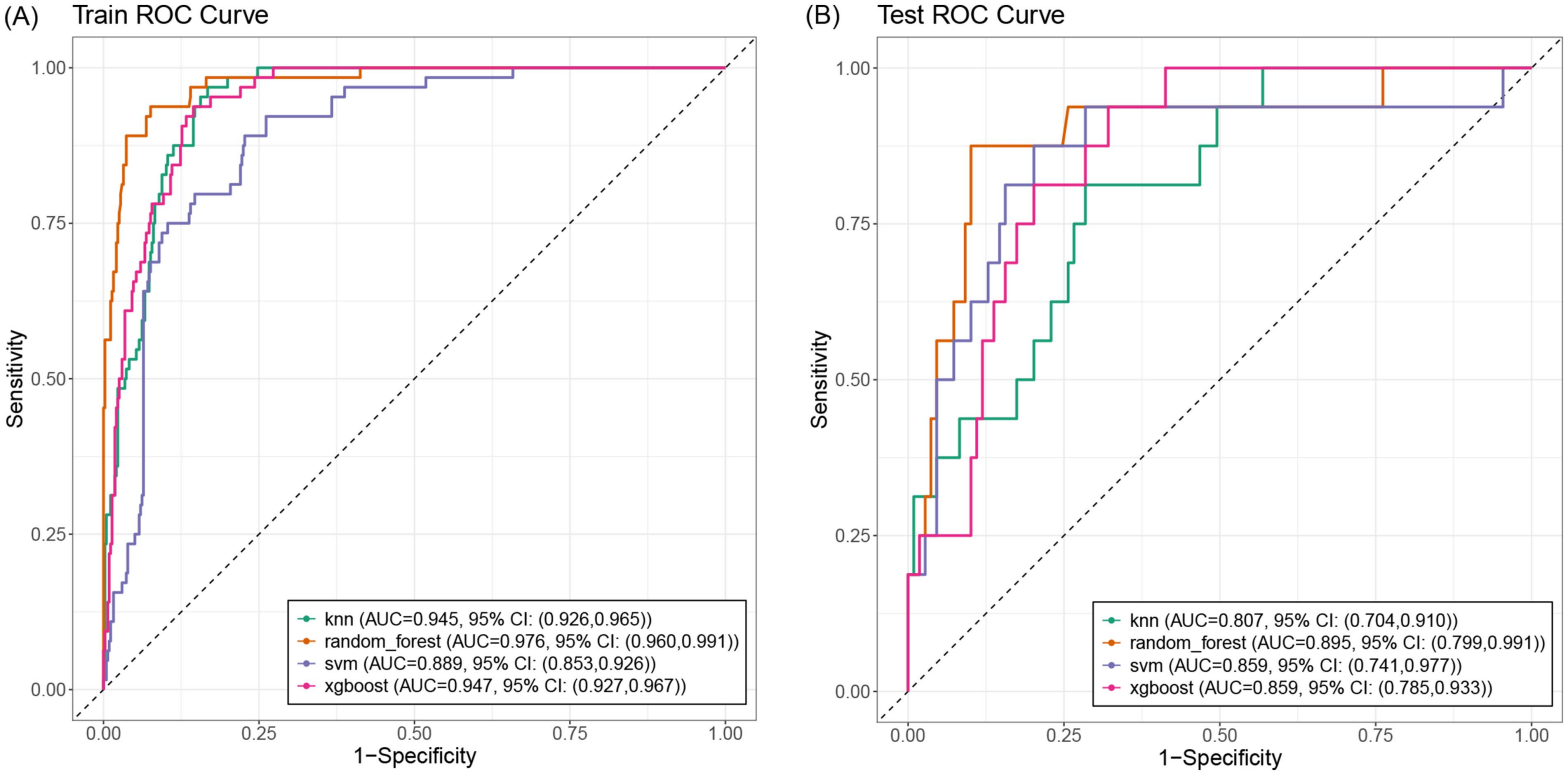
The ROC curves of different ML models in train **(A)** and test **(B)** set. The AUC of XGBoost, RF, SVM processed by upsampling. The AUC of KNN processed by downsampling.

**Figure 5:**
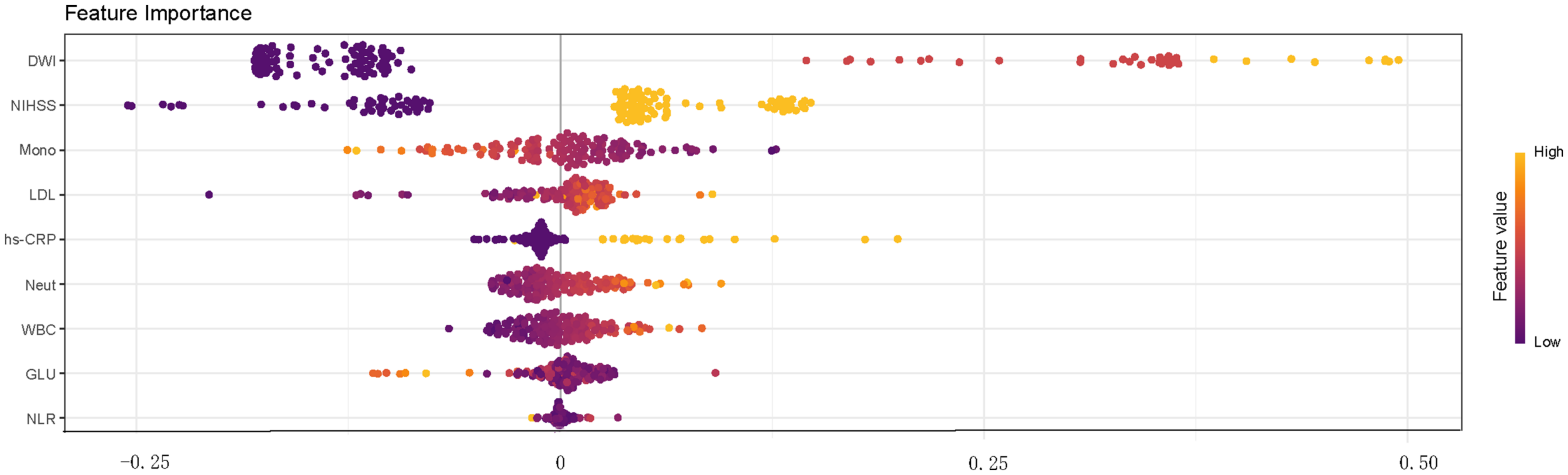
The importance ranking of different risk factors with stability and interpretation using SVM model processed by upsampling.

Through 10-fold stratified cross-validation and simulated annealing algorithm, the optimal sampling method for the AUC across four machine learning models was identified as follows: XGBoost, RF, and SVM achieved the best AUC values under upsampling, while KNN achieved the best AUC value under undersampling (Figure 3). The parameters for the four best machine learning models were shown in Supplementary Table S1. The optimal model among the four machine learning methods was selected as the SVM model that balanced the data through upsampling in the 16 workflows. In the training set and test set, XGBoost achieved AUC values of 0.947 (95% CI: 0.927, 0.967) and 0.859 (95% CI: 0.741, 0.977), RF achieved AUC values of 0.976 (95% CI: 0.960, 0.991) and 0.895 (95% CI: 0.941, 0.977), SVM achieved AUC values of 0.889 (95% CI: 0.853, 0.926) and 0.859 (95% CI: 0.741, 0.977), KNN achieved AUC values of 0.945 (95% CI: 0.926, 0.965) and 0.807 (95% CI: 0.704, 0.910) (Figure 4). In the training set, both the Random Forest and XGBoost models exhibited extremely high AUC values, necessitating vigilance against overfitting risks that might compromise the model’s generalization capability. In this study, we also compared the specificity, sensitivity, accuracy, positive predictive value (PPV), negative predictive value (NPV), precision, recall, F1 score, and AUC values of each model under the optimal sampling mode in the test set (Supplementary Table S2). The decision curve of the SVM model processed by upsampling showed that the model provided a significant net benefit compared to the baseline strategy when the threshold probability ranged from 0.001 to 0.476 in the training set. In the test set, the model also demonstrated a good net benefit, maintaining a high net benefit level within the threshold probability range of 0.017 to 0.485 (Supplementary Figure S1). SHAP plot analysis indicated that the top five most important features were DWI cerebral infarction area, NIHSS score at admission (0–2 points or 3–5 points), monocyte count, LDL level, and whether hs-CRP > 5 mg/L (Figure 5). These factors had a critical impact on early neurological deterioration after IVT in patients with mild stroke.

### Comparison of various logistic regression models

**Figure 6:**
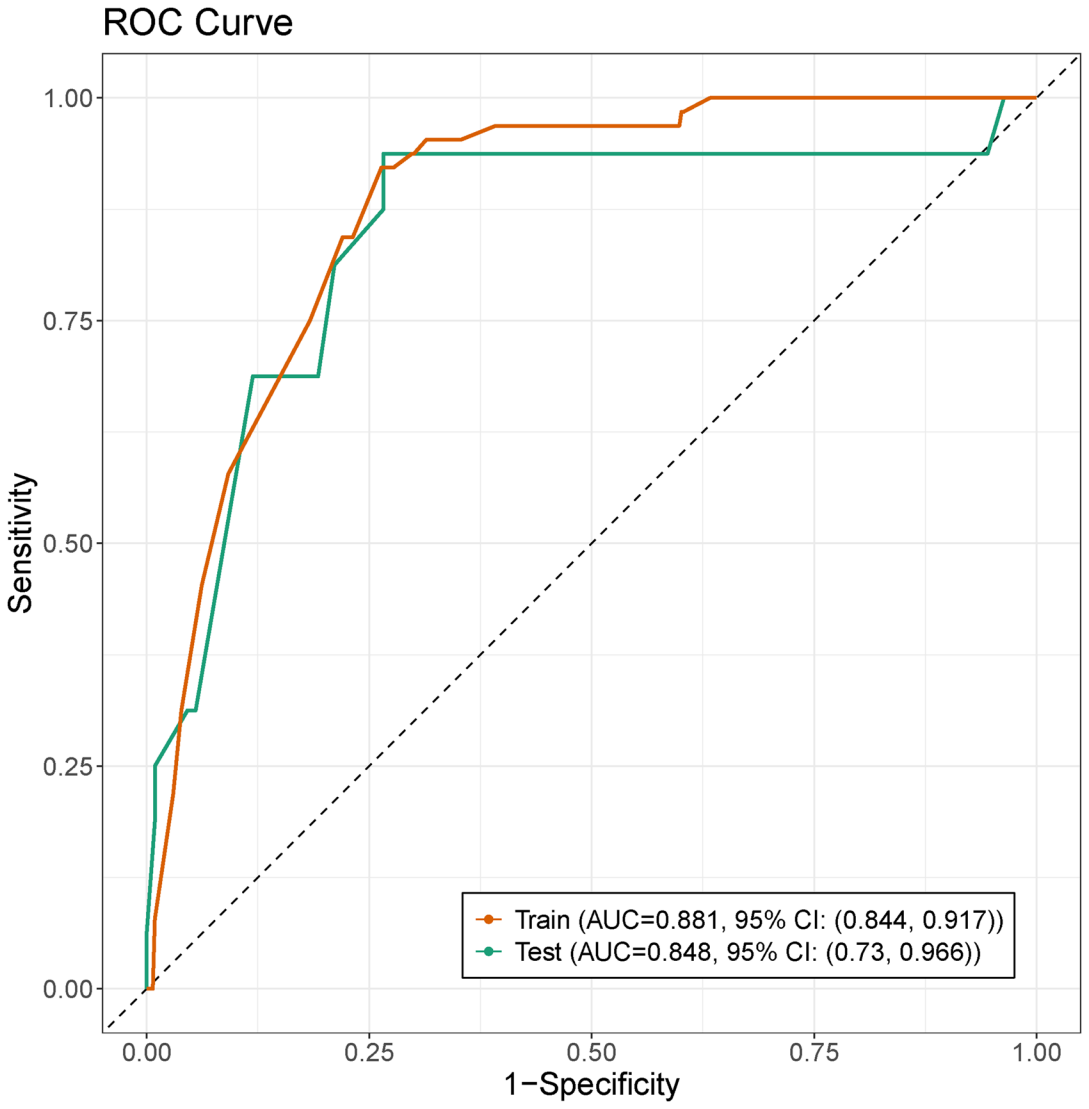
The ROC curves of Model m4 in train and test set.

**Figure 7:**
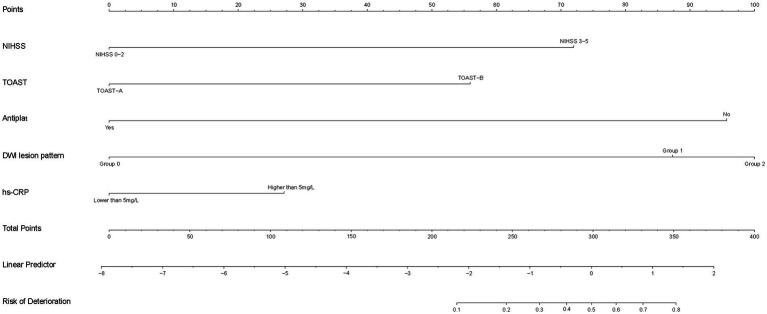
Nomogram for predicting early neurological deterioration after thrombolysis in patients with mild stroke.

Model m0 included the following variables: presence of coronary heart disease, prior use of antiplatelet agents, prior use of plaque-stabilizing agents, NIHSS score at admission (0–2 points or 3–5 points), TOAST type, white blood cell count, neutrophil count, FIB value, admission blood glucose level, LDL, DWI cerebral infarction area, and whether hs-CRP > 5 mg/L. Model m1 included all variables. Model m2 included variables selected through backward selection: age, prior use of antiplatelet agents, LDL level, TG, TG/HDL, and DWI cerebral infarction extent. Model m3 included variables selected through stepwise selection: gender, age, presence of coronary heart disease, LDL, TG, TG/HDL, DWI cerebral infarction area, neutrophil count, smoking status, prior use of plaque-stabilizing agents, whether hs-CRP > 5 mg/L, OTT and PT. After selecting the predictive variables with significant differences (*p* < 0.05) using the above method, the variables for model m4 were reconfigured: prior use of antiplatelet agents, NIHSS score at admission (0–2 points or 3–5 points), TOAST type, whether hs-CRP > 5 mg/L, and DWI cerebral infarction area. By comparing the *p*-values from the ANOVA test and the number of variables of the five models, model m4 was ultimately selected as the optimal logistic model. The AUC of this predictive model was 0.881 (95% CI, 0.844–0.917), with an internal validation AUC of 0.848 (95% CI, 0.73–0.966) (Figure 6). The confidence intervals for each variable in the m4 model were shown in Table 2. A nomogram was developed based on the optimal logistic model (Figure 7). The model had a sensitivity of 0.688 and specificity of 0.862 in the test set. The decision curve analysis showed that the nomogram to predict END after thrombolysis in patients with mild stroke would be beneficial when the threshold probability was 0.001–0.518 in the training set and 0.001–0.738 in the test set (Supplementary Figure S2).

**Table 2:** The multiple logistic regression analysis of risk factors for predicting early neurological deterioration after thrombolysis in the model m4.

Variables	Logistic regression	
	OR (95% CI)	*P*
NIHSS 3–5	6.660(2.540–22.900)	<0.001
TOAST-B	4.370(2.150–9.150)	<0.001
Antiplat-Yes	0.080(0.004–0.458)	0.021
DWI-Group 0	Ref	
DWI-Group 1	9.970(4.720–22.800)	<0.001
DWI-Group 2	13.900(5.460–37.600)	<0.001
CRP-higher than 5 mg/L	2.040(1.070–3.890)	0.029

## Discussion

Through machine learning model and logistic regression model analysis, it was found that the DWI cerebral infarction area, TOAST type, NIHSS score at admission (0–2 points or 3–5 points), LDL value, whether hs-CRP > 5 mg/L, monocyte value and prior use of antiplatelet agents might be the main factors affecting early neurological deterioration after thrombolysis in patients with mild stroke.

It was found that infarcts in the basal ganglia, periventricular regions, and brainstem were more likely to result in worsening neurological function after thrombolysis in patients with mild strokes in both machine learning and logistics regression models. Previous studies have also reported adverse outcomes in patients with deep white matter or basal ganglia infarcts following thrombolysis or endovascular therapy (9–11). This may be due to the fact that the basal ganglia, periventricular areas and brainstem regions are traversed by corticospinal tracts and are susceptible to damage owing to obstruction of collateral blood flow, thus making them more susceptible to motor dysfunction. Motor dysfunction is significantly associated with poor outcomes in mild stroke (3). Therefore, particular attention should be given to patients with perforating lesions and brainstem strokes, even if they exhibit only mild neurological deficits at presentation and after rt-PA therapy. We also found that mild stroke patients with NIHSS score of 3–5 points at onset were more likely to have early neurologic deterioration after thrombolysis. This was consistent with the study by Wang L et al. (12) Generally, patients with large vessel occlusion (LVO) often presented with relatively severe strokes, but approximately one-third of patients with LVO present with mild strokes at onset were characterized by low NIHSS scores (13). This type of patient is highly prone to progression in clinical settings (5, 14). For such mild stroke patients, we may be more inclined to administer thrombolysis. In the meantime, neurointerventional embolization needs to be ready. Additionally, we found that IVT treatment appears to be more effective for cardioembolic mild strokes. This result may be related to the composition of the thrombus. Thrombi in cardioembolic strokes contain more fibrin and platelets, while other thrombi contain more red blood cells. Rt-PA has a high affinity for fibrin, which may facilitate thrombus dissolution (15).

CRP is a non-specific inflammatory marker. Data indicated that extremely early elevation of CRP (approximately >7 mg/L) was helpful in stroke management; Extremely early elevation of CRP (average >20 mg/L) in ischemic and hemorrhagic strokes was a marker of poor clinical prognosis (CRP levels may be higher in the former); Additionally, elevated CRP detected within 72 h of ischemic stroke or transient ischemic attack was associated with an increased risk of stroke recurrence within 1 year, whereas no such association was observed when measured between 72 h and 8 days post-stroke (16–20). Several previous studies have demonstrated the important role of inflammation in first stroke onset, recurrent stroke, and stroke prognosis (21–23). Our study identified mild stroke patients with higher CRP who were more likely to experience early neurologic deterioration after thrombolysis. Meanwhile, in the feature importance ranking of SVM model, we found that mild stroke patients with higher neutrophil values, higher monocyte values and higher leukocyte values were also prone to early neurological deterioration after thrombolysis. This suggests that inflammation plays an important role in stroke progression. Inflammation therapy could be a new target for the future treatment of stroke (24).

We utilized artificial intelligence (AI) to predict early neurological deterioration after thrombolysis in patients with mild stroke based on risk factors identified from clinical data, and developed four mainstream machine learning models (KNN, SVM, XGBoost, and RF). We compared these four machine learning models and found that the SVM model performed better in this study. We also found that logistic regression model and machine learning model demonstrated comparable performance in this single-center retrospective dataset. Compared to some of the machine learning algorithms in our study, the advantages of logistic regression may be attributed to several factors. First, the logistic regression model achieved a good AUC value with five variables while avoiding overfitting of the data. Second, the interpretability of logistic regression allowed for more effective feature selection and model improvement, ensuring that only clinically relevant predictors were included, whereas machine learning models typically prioritized statistical patterns over treatment relevance. Finally, the absence of highly interactive or nonlinear effects in our dataset might reduce the advantages of machine learning algorithms, which were typically adept at capturing such complex relationships. In datasets lacking complex interactions or large sample sizes, their advantages were diminished (25).

In this study, first, we further improved the accuracy of the predictive model for early neurological deterioration after thrombolysis in patients with mild stroke by incorporating relevant imaging examinations. Second, we established different machine learning models and logistic regression models. By comparing them, the optimal model was obtained. Finally, we used artificial intelligence to predict early neurological deterioration after IVT in patients with mild stroke and proposed a solution, paving the way for the development of AI-driven tools.

However, this study also had several limitations: first, the small sample size and relatively unbalanced data restricted the statistical power of the machine learning model, which might also affect the model’s accuracy. Second, as this study was a single-center retrospective analysis, the lack of external validation limited the generalizability of the findings. Furthermore, the long-term prognosis of patients with mild stroke undergoing thrombolysis was not further evaluated in this study. Finally, In this study, some head MRI and cranial-neck CTA results were obtained 24–48 h after admission for thrombolysis. Although some hospitals can now complete these examinations within 24 h post-thrombolysis, the uncertainty in the timing of these tests still led to data leakage. This caused model distortion, compromising the model’s clinical utility and authenticity.

## Conclusion

Despite these limitations, our study results suggested that by focusing on easily obtainable variables, including DWI cerebral infarction area, TOAST type, NIHSS score at admission (0–2 points or 3–5 points), LDL value, whether hs-CRP > 5 mg/L, monocyte value and antiplatelet agents, the risk for END in patients with mild stroke undergoing thrombolysis could be predicted. We also found that logistic regression model and machine learning model demonstrated comparable performance in this single-center retrospective dataset.

## Data Availability

The raw data supporting the conclusions of this article will be made available by the authors, without undue reservation.

## Supplementary material

SUPPLEMENTARY FIGURE S1 Decision curve analysis (DCA) of SVM model processed by upsampling in train **(A)** and test **(B)** set.

SUPPLEMENTARY FIGURE S2 Decision curve analysis (DCA) of Model m4 in train **(A)** and test **(B)** set.

## References

[1] Saber H Khatibi K Szeder V Tateshima S Colby GP Nour M . Reperfusion therapy frequency and outcomes in mild ischemic stroke in the United States. Stroke. (2020) 51:3241–9. doi: 10.1161/strokeaha.120.030898, 33081604

[2] Mazya MV Cooray C Lees KR Toni D Ford GA Bar M . Minor stroke due to large artery occlusion. When is intravenous thrombolysis not enough? Results from the SITS international stroke thrombolysis register. Eur Stroke J. (2018) 3:29–38. doi: 10.1177/2396987317746003, 31008335 PMC6453245

[3] Strambo D Zambon AA Roveri L Giacalone G Di Maggio G Peruzzotti-Jametti L . Defining minor symptoms in acute ischemic stroke. Cerebrovasc Dis. (2015) 39:209–15. doi: 10.1159/000375151, 25791530

[4] Li C Li J Cao Z. A nomogram prediction model for poor outcome in patients with minor ischemic stroke. Int J Cerebrovasc Dis. (2024) 32:241–6. doi: 10.3760/cma.j.issn.1673-4165.2024.04.001

[5] Kim JM Moon J Ahn SW Shin HW Jung KH Park KY. The etiologies of early neurological deterioration after thrombolysis and risk factors of ischemia progression. J Stroke Cerebrovasc Dis. (2016) 25:383–8. doi: 10.1016/j.jstrokecerebrovasdis.2015.10.010, 26597264

[6] Powers WJ Rabinstein AA Ackerson T Adeoye OM Bambakidis NC Becker K . Guidelines for the early management of patients with acute ischemic stroke: 2019 update to the 2018 guidelines for the early Management of Acute Ischemic Stroke: a guideline for healthcare professionals from the American Heart Association/American Stroke Association. Stroke. (2019) 50:e344. doi: 10.1161/str.0000000000000211, 31662037

[7] Chinese Society of Neurology, Chinese Stroke Society. Chinese guidelines for diagnosis and treatment of acute ischemic stroke 2023. Chin J Neurol. (2024) 57:523–59. doi: 10.3760/cma.j.cn113694-20240410-00221

[8] Brott T Adams HP Jr Olinger CP Marler JR Barsan WG Biller J . Measurements of acute cerebral infarction: a clinical examination scale. Stroke. (1989) 20:864–70. doi: 10.1161/01.str.20.7.864, 2749846

[9] Liu D Scalzo F Starkman S Rao NM Hinman JD Kim D . DWI lesion patterns predict outcome in stroke patients with thrombolysis. Cerebrovasc Dis. (2015) 40:279–85. doi: 10.1159/000441153, 26513397 PMC4955553

[10] Rosso C Colliot O Valabrègue R Crozier S Dormont D Lehéricy S . Tissue at risk in the deep middle cerebral artery territory is critical to stroke outcome. Neuroradiology. (2011) 53:763–71. doi: 10.1007/s00234-011-0916-5, 21789602

[11] Kim DH Lee DS Nah HW Cha JK. Clinical and radiological factors associated with unfavorable outcome after intravenous thrombolysis in patients with mild ischemic stroke. BMC Neurol. (2018) 18:30. doi: 10.1186/s12883-018-1033-4, 29544461 PMC5856376

[12] Wang L Li G Hao Y Hao M Xiong Y. Intravenous thrombolysis for mild stroke: NIHSS 3-5 versus NIHSS 0-2. J Stroke Cerebrovasc Dis. (2023) 32:107070. doi: 10.1016/j.jstrokecerebrovasdis.2023.107070, 36905743

[13] Kim JT Heo SH Yoon W Choi KH Park MS Saver JL . Clinical outcomes of patients with acute minor stroke receiving rescue IA therapy following early neurological deterioration. J Neurointerv Surg. (2016) 8:461–5. doi: 10.1136/neurintsurg-2015-011690, 25910943

[14] Park TH Lee JK Park MS Park SS Hong KS Ryu WS . Neurologic deterioration in patients with acute ischemic stroke or transient ischemic attack. Neurology. (2020) 95:e2178–91. doi: 10.1212/wnl.0000000000010603, 32817184

[15] Vaclavik D Vilionskis A Jatuzis D Karlinski MA Gdovinova Z Kõrv J . Clinical outcome of cardioembolic stroke treated by intravenous thrombolysis. Acta Neurol Scand. (2018) 137:347–55. doi: 10.1111/ane.12880, 29218699

[16] Zhang X Wang A Zhang J Singh M Liu D Zuo Y . Association of plasma C-reactive protein with ischaemic stroke: a mendelian randomization study. Eur J Neurol. (2020) 27:565–71. doi: 10.1111/ene.14113, 31692152

[17] Erdal GS Hursitoglu M Erdogan HA Yildirim G Yayla V Issever H . Serum C-reactive protein and sex hormone levels in the early hyperacute phase of stroke. Clin Lab. (2021) 67:67. doi: 10.7754/Clin.Lab.2020.200610, 33616319

[18] Akhter S Das SN Sutradhar SR Basher MS Khan MK. Level of serum C-reactive protein among patients with stroke. Mymensingh Med J. (2018) 27:461–6. 30141432

[19] Mazaheri S Reisi E Poorolajal J Ghiasian M. C-reactive protein levels and clinical outcomes in stroke patients: a prospective cohort study. Arch Iran Med. (2018) 21:8–12. 29664664

[20] Wang Y Li J Pan Y Wang M Meng X Wang Y. Association between high-sensitivity C-reactive protein and prognosis in different periods after ischemic stroke or transient ischemic attack. J Am Heart Assoc. (2022) 11:e025464. doi: 10.1161/jaha.122.025464, 35766270 PMC9333386

[21] Kaptoge S Di Angelantonio E Lowe G Pepys MB Thompson SG Collins R . C-reactive protein concentration and risk of coronary heart disease, stroke, and mortality: an individual participant meta-analysis. Lancet. (2010) 375:132–40. doi: 10.1016/s0140-6736(09)61717-7 20031199 PMC3162187

[22] McCabe JJ O'Reilly E Coveney S Collins R Healy L McManus J . Interleukin-6, C-reactive protein, fibrinogen, and risk of recurrence after ischaemic stroke: systematic review and meta-analysis. Eur Stroke J. (2021) 6:62–71. doi: 10.1177/2396987320984003, 33817336 PMC7995315

[23] Huang YW Yin XS Li ZP. Association of the systemic immune-inflammation index (SII) and clinical outcomes in patients with stroke: a systematic review and meta-analysis. Front Immunol. (2022) 13:1090305. doi: 10.3389/fimmu.2022.1090305, 36591305 PMC9797819

[24] Kelly PJ Lemmens R Tsivgoulis G. Inflammation and stroke risk: a new target for prevention. Stroke. (2021) 52:2697–706. doi: 10.1161/strokeaha.121.034388, 34162215

[25] Lo Vercio L Amador K Bannister JJ Crites S Gutierrez A MacDonald ME . Supervised machine learning tools: a tutorial for clinicians. J Neural Eng. (2020) 17:17. doi: 10.1088/1741-2552/abbff2, 33036008

