# Supplementary figures and images for: Comparison of logistic regression and machine learning methods for predicting early neurological deterioration after thrombolysis in patients with mild stroke
Source: Front Neurol. 2026 Mar 4;17:1703890. doi: 10.3389/fneur.2026.1703890 (PMC12996063; doi:10.3389/fneur.2026.1703890)

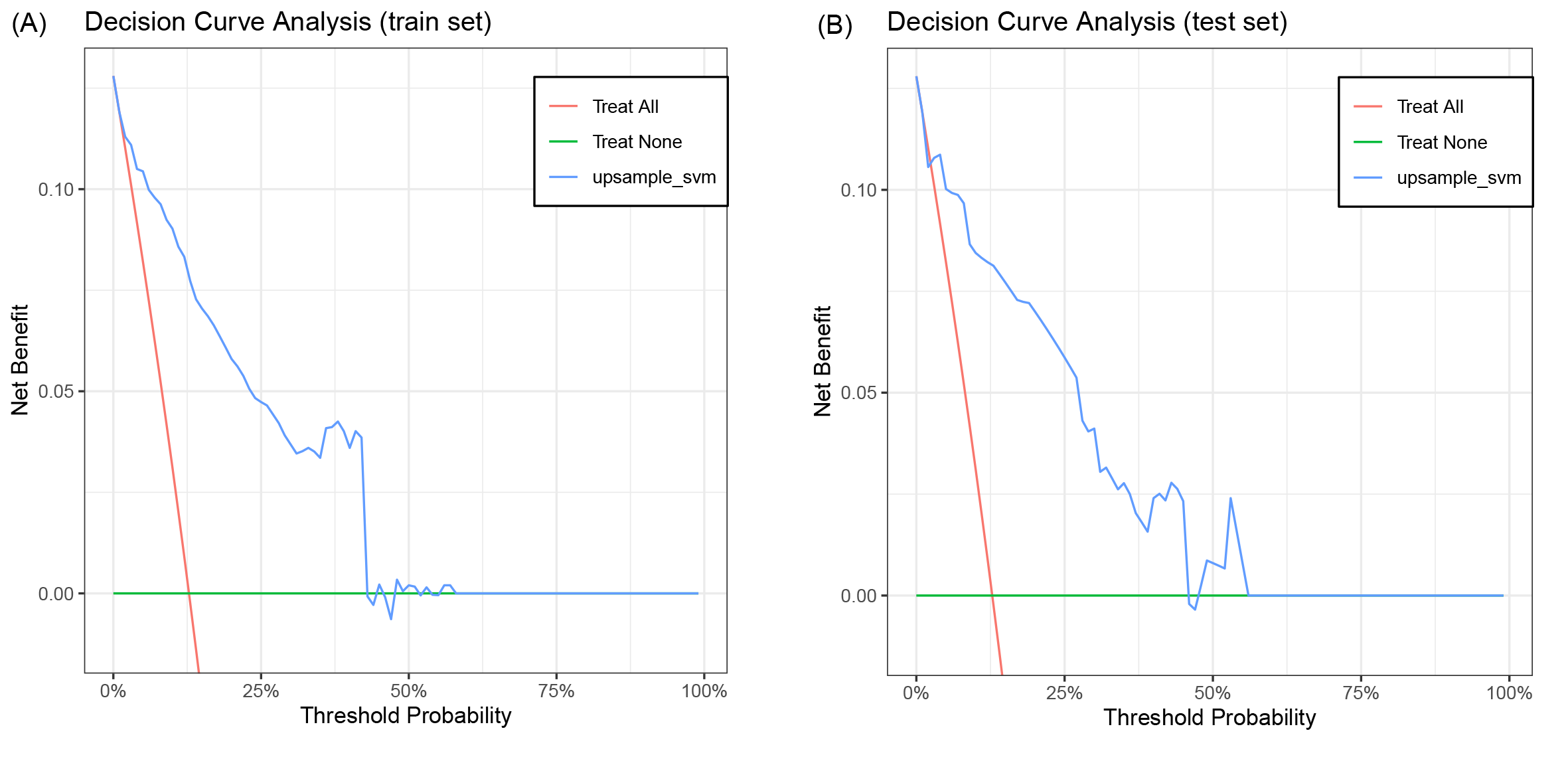

Supplement: SUPPLEMENTARY FIGURE S1 — Decision curve analysis (DCA) of SVM model processed by upsampling in train (A) and test (B) set. [file Image_1.tif]

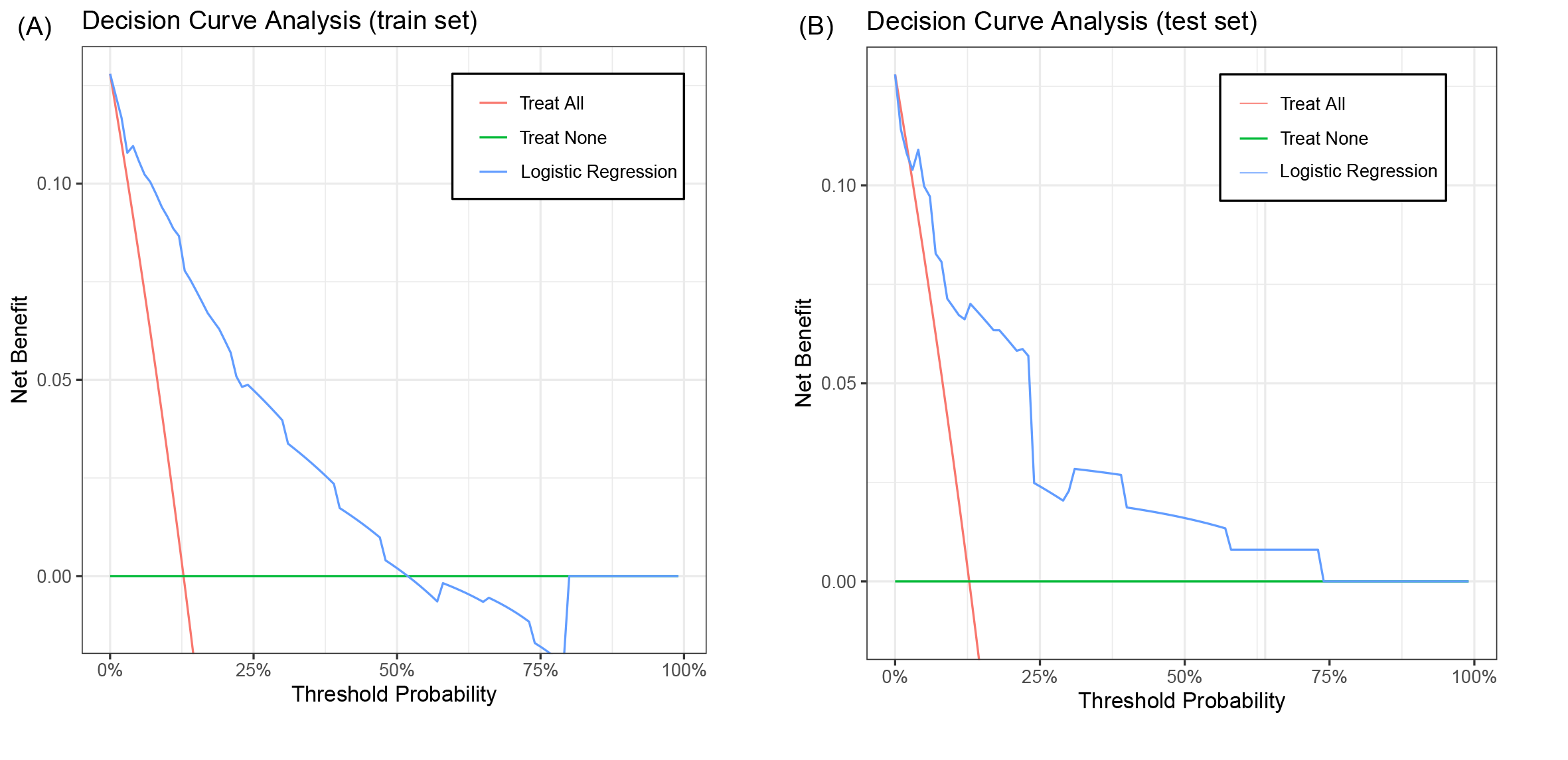

Supplement: SUPPLEMENTARY FIGURE S2 — Decision curve analysis (DCA) of Model m4 in train (A) and test (B) set. [file Image_2.tif]
