# Supplementary material for: Comparison of logistic regression and machine learning methods for predicting early neurological deterioration after thrombolysis in patients with mild stroke
Source: Front Neurol. 2026 Mar 4;17:1703890. doi: 10.3389/fneur.2026.1703890 (PMC12996063; doi:10.3389/fneur.2026.1703890)
Supplement: Supplementary file 3 [file Table_1.doc]

Table S1：Hyperparameters of machine learning models

| Models | Hyperparameters |
| --- | --- |
| Random forest  upsample | mtry=0.505 trees=1567 min_n=11  over_ratio=0.929 threshold=0.788 |
| SVM  upsample | cost=2.629 rbf_sigma=0.025 margin=0.182 over_ratio=0.935 threshold=0.775 |
| XGBoost  upsample | mtry=0.920 trees=269 min_n=17 tree_depth=4 learn_rate=0.006 loss_reduction=5.081e-10 over_ratio=0.985 threshold=0.068 |
| KNN  downsample | knn_neighbors=15 weight_func=biweight dist_power=0.686  under_ratio=1.160 threshold=0.649 |
