# Supplementary material for: Comparison of logistic regression and machine learning methods for predicting early neurological deterioration after thrombolysis in patients with mild stroke
Source: Front Neurol. 2026 Mar 4;17:1703890. doi: 10.3389/fneur.2026.1703890 (PMC12996063; doi:10.3389/fneur.2026.1703890)
Supplement: Supplementary file 4 [file Table_2.doc]

| Table S2: Comparison the parameters of models for prediction of osteoporosis in the test set | | | | | | | | | |
| --- | --- | --- | --- | --- | --- | --- | --- | --- | --- |
| **Model** | **Sensitivity** | **Specificity** | **Pos Pred Value** | **Neg Pred Value** | **Accuracy** | **Precision** | **Recall** | **F1** | **AUC** |
| KNN downsample | 0.625 | 0.761 | 0.278 | 0.933 | 0.744 | 0.278 | 0.625 | 0.385 | 0.807 |
| Random forest upsample | 0.875 | 0.881 | 0.519 | 0.980 | 0.880 | 0.519 | 0.875 | 0.651 | 0.895 |
| SVM upsample | 0.813 | 0.826 | 0.406 | 0.968 | 0.824 | 0.406 | 0.813 | 0.542 | 0.859 |
| XGBoost upsample | 0.750 | 0.826 | 0.387 | 0.957 | 0.816 | 0.387 | 0.750 | 0.511 | 0.859 |
